# Genetic and environmental risk of congenital anomaly

**DOI:** 10.2478/abm-2022-0031

**Published:** 2023-08-01

**Authors:** Editorial Office of Asian Biomedicine

**Affiliations:** Faculty of Medicine, Chulalongkorn University, Bangkok 10330, Thailand

Estimates of the prevalence of congenital anomalies vary due to the incompleteness of data and the variety of methodologies used [1]. Both genetic defects and/or environmental exposures are considered to be associated with congenital anomalies [1, 2, 3, 4]. In order to understand the true nature and extent of the conditions in various parts of the world, the collection of data on the prevalence, spectrum, trend, and outcomes of congenital anomalies from different countries needs some standardization. In many countries, the termination of pregnancy once congenital anomalies are detected has obscured the true prevalence of the condition and obscures the need for effective prevention [1].

In a systematic study from North India, the International Statistical Classification of Diseases and Related Health Problems (ICD) and a standardized surveillance tool such as the World Health Organization (WHO) Birth Defects Surveillance Manual were used to estimate the prevalence, spectrum, trend, and outcomes of congenital anomalies over time [5]. The study found that the overall prevalence of anomalies was 182 (95% confidence interval [CI]: 173–191) per 10,000 live births [5]. Common congenital anomalies included malformation of the circulatory, musculoskeletal, and urinary systems [5]. No significant trend was observed in the annual prevalence, individual malformations, or contribution of congenital anomalies to overall mortality over a period of 2 decades.

Both environment and genetic associations have played an important role in the occurrence of congenital anomalies. Congenital anomalies including congenital heart diseases, cleft lip, and cleft palate have been imputed to maternal exposure to air pollution, toxic chemicals, and parental smoking [6]. Maternal conditions such as infections during pregnancy, pregestational and gestational diabetes mellitus, maternal obesity, and maternal drug intake have also been shown to play an important role [6]. Maternal folic acid-and-iron supplementation has a preventive effect on congenital heart defects [6]. The genetic loci associated with congenital anomalies include the genes for methylene tetrahydrofolate reductase (MTHFR), methionine synthase reductase (MTRR), methionine synthase (MTR), GATA binding protein 4 (GATA4), NK2 homeobox 5 (NKX2-5), steroid 5 alpha-reductase type 2 (SRD5A2), cystic fibrosis transmembrane conductance regulator (CFTR), and single-nucleotide polymorphisms at 1p22 and 20q12 [6]. Neural tube defects, which are considered a common congenital malformation affecting the development of the central nervous system, are thought to be associated with both genetic and environmental factors [7]. Some neural tube defects are prevented by folic acid supplementation [8], as advocated by the WHO [9, 10, 11, 12] and implemented in some countries [13, 14].

In this volume, Naeem et al. [15] describe the burden of congenital and hereditary anomalies in the war-affected territory at the Pakistan–Afghanistan border. They report a high incidence of neurological, sensorineural, and limb defects. There has been a relatively low level of parental consanguinity. The study suggests an important contribution of nongenetic factors. A thorough evaluation of these conditions will require a comprehensive history, physical examination, and appropriate testing to reduce unwanted consequences to the affected. The prenatal history can uncover specific exposures and etiologic factors. In addition to a complete family history, such as parental age, associated diseases, drug intake, exposure to chemicals, and consanguinity, a complete pedigree (four generations, if possible) should be obtained to help uncover genetic causes. Ethnic and racial origin may reveal important association with the anomalies [16].

In conclusion, a standardized surveillance system using common tools is necessary to enable comparability and reveal variations in the prevalence of congenital anomalies and their associated risk factors in various regions of the world. Some risk factors, such as those associated with maternal behaviors, environmental factors, and potential genetic risks, may be documented. Some risk factors such as folic acid deficiencies can be readily corrected. Other risk factors such as racial and ethnic disparities may be managed through other effective national, regional, and local policies and plans.
